# Travel Mode and Physical Activity at Sydney University

**DOI:** 10.3390/ijerph10083563

**Published:** 2013-08-09

**Authors:** Chris Rissel, Corinne Mulley, Ding Ding

**Affiliations:** 1 Prevention Research Collaboration, Sydney School of Public Health, University of Sydney, NSW 2006, Australia; E-Mail: melody.ding@sydney.edu.au; 2 Institute of Transport and Logistic Studies, University of Sydney, NSW 2006, Australia; E-Mail: corinne.mulley@sydney.edu.au

**Keywords:** physical activity, active travel, commuting, university

## Abstract

How staff and students travel to university can impact their physical activity level. An online survey of physical activity and travel behaviour was conducted in early November 2012 to inform planning of physical activity and active travel promotion programs at the University of Sydney, Australia as part of the “Sit Less, Move More” sub-committee of the Healthy University Initiative, and as baseline data for evaluation. There were 3,737 useable responses, 60% of which were from students. Four out of five respondents travelled to the University on the day of interest (Tuesday, November 30, 2012). The most frequently used travel modes were train (32%), car as driver (22%), bus (17%), walking (17%) and cycling (6%). Staff were twice as likely to drive as students, and also slightly more likely to use active transport, defined as walking and cycling (26% *versus* 22%). Overall, 41% of respondents were sufficiently active (defined by meeting physical activity recommendations of 150 min per week). Participants were more likely to meet physical activity recommendations if they travelled actively to the University. With a high proportion of respondents using active travel modes or public transport already, increasing the physical activity levels and increasing the use of sustainable travel modes would mean a mode shift from public transport to walking and cycling for students is needed and a mode shift from driving to public transport or active travel for University staff. Strategies to achieve this are discussed.

## 1. Introduction

Workplace travel plans have the potential to promote physical activity through active travel options (walking, cycling, public transport and combinations of these modes of travel) and at the same time address organisational concerns such as environmental impact, traffic congestion and parking pressures [1]. Active transport has many co-benefits, including positive impacts on climate change and sustainability [2]. Workplace travel plans are behaviour change interventions designed to increase uptake of sustainable transport modes for commuting and business trips, often at the expense of car driving. They have been deployed extensively throughout Australia, the United States, Canada, the Netherlands and the UK [3,4,5], but not necessarily as part of routine workplace health promotion programs, nor at Universities [6]. They typically involve a survey of travel behaviour to understand existing travel behaviours [7]. A recent report on the cost-effectiveness of prevention programs highlighted that an optimal mix of cost-effective interventions for increasing physical activity at the population level could include travel planning [8].

There are many examples of travel planning projects demonstrating changes in travel modes, where broad coverage of the population has been targeted for minimal capital outlay demonstrating clear economic and financial returns, as well as large projected reductions in car use and carbon emissions [9,10,11,12,13]. However, there is limited Australian evidence on whether workplace travel plans can improve employee health [14]. There are few evaluations of workplace travel plans published in the peer reviewed literature [1,14], despite clear indications that active commuting is associated with a range of positive health outcomes [15]. These benefits include increased cardiovascular health [13,16,17] and weight loss/maintenance of healthy weight [18,19].

Universities, as a workplace focused on research and new ideas, might reasonably be expected to embrace the concept of travel planning, including provision for active commuters. A survey of active travel at The University of Western Australia found 21.5% of staff and 46.8% of students regularly used active modes (including public transport), and potentially an additional 30% of staff and students would switch to active modes under some circumstances [20]. However, overall physical activity was not assessed in this study. A later study at the University of Western Australia found that, compared with private motor vehicle users, public transport users performed more daily steps, and the odds of achieving 10,000 steps/day was higher in public transport users compared with private motor vehicle users after adjusting for gender, age group and average daily minutes of self-reported leisure-time physical activity [21]. However, the contribution of walking and cycling to overall physical activity was not considered in this study. Furthermore, a mid-western US college reported that individual active travel efficacy factors were most important for explaining active travel to campus [22], but small college towns are not comparable with large cosmopolitan cities in terms of transportation infrastructure and travel patterns.

In New South Wales (NSW), the most populous state in Australia, the prevalence of walking to work among employed respondents from 2005 to 2010 ranged between 5.1–7.3%, and 1.4–1.8% for cycling [23]. Walking and cycling is more common in the inner city areas of Sydney compared with outer suburbs [24,25], with cycling in inner Sydney representing 2.2% of journeys to work, and walking representing 10.1% of journeys to work. In 2011 67.7% of all employees in the Greater Sydney Statistical Area drove to work, and 23.2% used public transport, although in the Sydney Central Business District 70% of employees used the train or bus, and 41.4% in Redfern (nearest train station to the University of Sydney) used the train or bus [26].

The University of Sydney is one of the oldest and largest Universities in Australia. It is currently developing a “Healthy University” program. As part of a focus on encouraging greater physical activity, this program has become linked with the way in which students and staff travel regularly to the University campuses as a way of integrating travel planning and health goals. This paper describes a University-wide survey of travel behaviour and physical activity. The survey outlined in this paper was designed to inform the development of a Green Travel Plan (reducing the Universities carbon footprint and increasing active travel) and to provide a physical activity baseline for the Healthy Sydney University program. It is the first Australian study to describe the links between physical activity outcomes and travel behaviour (including active travel) in a university context. A secondary research question is to examine and compare two definitions of active travel (with and without public transport use) in relation to achieving sufficient amount of physical activity. Such special consideration of public transport use in the context of active travel is important as public transport use has been found to contribute to overall physical activity [27,28].

## 2. Method

An on-line survey was developed to be delivered to all staff and students at the University of Sydney. An email containing a hyperlink to the on-line questionnaire was sent on behalf of the Vice-Chancellor to all students and staffs using existing University electronic communication networks. Student and staff organisations were also encouraged to participate in the survey. All data entered online by respondents were automatically collated into a database ready for data cleaning and analysis.

The survey focused on travel to the University on a specific Tuesday, which is considered a “typical” travel day. This is the approach used by the Australian Bureau of Statistics to collect census data about the journey to work, and is robust when used with a large sample. The travel day for the survey was Tuesday 30 October 2012. The weather on that day was a typical October day in Sydney with temperatures 14–23 °C with no rain [29]. The email from the Vice-Chancellor was distributed on the morning of the Thursday following (November 1). Respondents were asked if they travelled to the University on 30 October, and if they did, which campus they went to and what mode of travel they used. The travel mode question was “How did you travel to campus on Tuesday? Select the mode used for the longest part of your trip.” Respondents were given choices from motor vehicle travel (separately identifying car as driver, car as passenger, motorcycle and taxi), public transport train, bus, ferry or light rail) and active travel (including walk, bicycle, skateboard and non-motorised scooter).

Questions from the previously validated International Physical Activity Questionnaire (IPAQ) were included in the questionnaire to quantify physical activity in the past 7 days [30]. The total number of minutes of physical activity in the last week was calculated from the responses as well as identifying if respondents had achieved the recommended amount of weekly physical activity for health benefits (*i.e*., 150 min of moderate-to-vigorous physical activity). Outliers for total weekly physical activity were truncated to 960 min, as per protocols.

The questionnaire sought demographic information, including age, sex, highest educational achievement and residential postcode, staff or student status, and the Faculty within the University of Sydney in which they were located.

The analyses reported here describe travel patterns and self-reported physical activity levels for staff and student respondents. The distribution of students by Faculty, and sex distribution of staff and students have been compared with that reported in The University of Sydney 2011 Annual report [31] for establishing the representativeness of the survey sample to the target population using a chi square test. Mean total minutes of physical activity by travel mode were calculated. Logistic regression was used to calculate the adjusted odds of being an active traveller to the University in which the active transport definition was set to include the public transport traveller because of the need to undertake active travel for its access and egress and excluding public transport travellers for the more frequently cited definition of active travel. The research was approved by the Human Research Ethics Committee, The University of Sydney (Protocol No: 15206).

## 3. Results

Despite over 5,000 attempts to complete the questionnaire (n = 5,193), there was a relatively high number of incomplete surveys resulting from the exceptionally large load on the University computing system leading to system crashes leading to users having to re-start the survey. Surveys that were incomplete or were obviously duplicates were excluded from the analysis, leaving 3,737 usable responses (72%).

**Figure 1:**
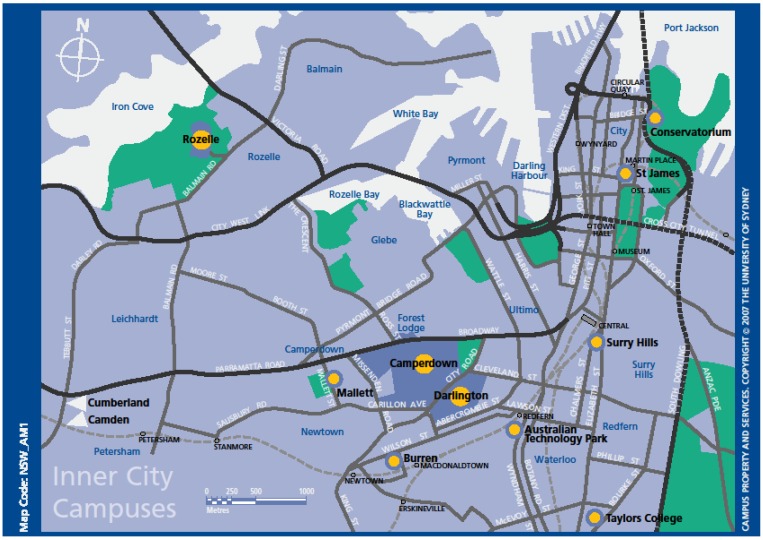
Inner city campuses of the University of Sydney *.

Sixty per cent of respondents were students (n = 2174), and the remainder staff or affiliates of the University. Four out of five of all respondents (79.9%) travelled to the main University Darlington/Camperdown campus close to the CBD in Sydney, Australia. Figure 1 shows the inner city Sydney University campuses and the location of the main Darlington/Camperdown campus which is 3 km from the Sydney central business district. Land use zoning is mixed, with some vibrant communities in the 5 km around the main campus. Topography is reasonably flat, but motor vehicle traffic can be heavy with major arterial roads through the area. Three quarters of respondents travelled to the University on the date specified, with more staff travelling to the University (84.5%) than students (72.2%) (see Table 1). Of respondents travelling to the Darlington/Camperdown campus approximately 40% lived within 5 km of the campus and 39% lived between 5 and 15 km from the University, based on their postcode of residence.

The distribution of students participating in the on-line survey differed to the distribution of students enrolled in the 16 major Faculties of the University (*p* < 0.001) as reported in the 2011 University of Sydney Annual Report [31]. Specifically, there were fewer students from the Business School and Health Sciences in our sample, and more students from the Science and Medical School faculties. There were more female student survey participants (72.2%) compared with enrolled female students at the University (57.3%) (*p* < 0.001). There were also more female staff survey participants (63.9%) than identified in the University Annual Report (54.8%) (*p* < 0.001).

* see http://sydney.edu.au/about/campuses/index.shtml for more information about location of University of Sydney campuses.

### 3.1. Travel Behaviour

The most frequently used travel modes were train (32%), car as driver (22%), bus (17%), walking (17%) and cycling (6%) (see Table 2). The car driving and active modes (walking and cycling) were higher, and public transport use was lower, than for the Sydney Central Business District which has a 74% public transport mode share and 7.9% active travel [32].

When travel mode is examined by staff/student status and campus, staff were twice as likely as students to drive a motor car to the University, and students more likely to use public transport (see Table 3). Staff were slightly more likely than students to cycle or walk.

### 3.2. Car Use

Of those people who drove to a campus (n = 651), 88.5% drove by themselves, and a further 10.5% had a second person with them. About half (53.1%) parked on campus with a permit, and a further 23.2% parked for free off-campus. One in ten (10.6%) parked for free on-campus. Car users were more likely to be a staff member (62.1%), be aged 25–44 years (40.9%) or age over 45 years (35.3%) and have a degree or higher (72.1%).

**Table 1:** Description of respondents.

Characteristic of respondent	Staff		Students		Total	
**Age:**	n	%	n	%	n	%
under 25 years	44	3.1	1,382	63.9	1,426	39.8
25–34 years	364	25.6	512	23.7	876	24.45
35–44 years	400	28.2	129	6.0	529	14.76
45–54 years	341	24.0	87	4.0	428	11.95
55–64 years	221	155.6	40	1.9	261	7.28
65 years or over	50	3.5	13	0.6	63	1.76
**Sex:**						
Male	513	36.1	602	27.8	1,115	31.11
Female	908	63.9	1,561	72.2	2,469	68.89
**Travelled to University**						
Yes	1,228	84.5	1,569	72.2	2,896	77.58
No	222	15.3	514	23.6	740	19.82
Live on campus	4	0.3	91	4.2	97	2.6
**Faculty or organisation**						
University-wide administration or services	300	21.3	0	0	300	8.42
Agriculture, Food and Natural Resources	26	1.9	24	1.1	50	1.4
Architecture, Design and Planning	15	1.1	36	1.7	51	1.43
Arts and Social Sciences	123	8.8	375	17.4	498	13.98
Business School	64	4.6	168	7.8	232	6.51
Dentistry	13	0.9	16	0.7	29	0.81
Education and Social Work	30	2.1	128	5.9	158	4.43
Engineering and Information Technologies	55	3.9	162	7.5	217	6.09
Health Sciences	98	7.0	291	13.5	389	10.92
Pharmacy	15	1.1	68	3.2	83	2.33
Science	164	11.7	402	18.6	566	15.89
Sydney College of the Arts	13	0.9	27	1.3	40	1.12
Sydney Conservatorium of Music	13	0.9	31	1.4	44	1.23
Sydney Law School	26	1.9	89	4.1	115	3.23
Sydney Medical School	309	22.0	202	9.4	511	14.34
Sydney Nursing School	23	1.6	53	2.5	76	2.13
Veterinary Science	33	2.4	77	3.6	110	3.09

### 3.3. Bicycle Use

Of 186 bicycle users (6.2% of travel mode) the majority parked their bicycles in a University designated bicycle rack (61.8%), with a further 30% parking their bicycle inside an office or workspace. Bicycle riders were more likely to be male (58.7%), be a staff member (56.4%), be aged 25–44 years (62.8%), and have a degree (50.9%).

**Table 2:** Travel mode to University campuses.

	Staff		Students		Total	
**Travel mode**	N	%	n	%	N	%
Car as driver	405	33.0	217	13.8	651	22.48
Car as passenger	39	3.2	55	3.5	97	3.35
Motorcycle	15	1.2	6	0.4	21	0.73
Train	280	22.8	612	39.0	921	31.8
Bus	157	12.8	325	20.7	496	17.13
Ferry	6	0.5	3	0.2	10	0.35
Light rail	0	0	1	0.1	1	0.03
Walk	217	17.7	258	16.4	490	16.92
Bicycle	101	8.2	78	5.0	186	6.42
Taxi	1	0.1	3	0.3	4	0.14
Skateboard	0	0	1	0.1	1	0.03
Scooter (no motor)	0	0	2	0.1	2	0.07
Other	7	0.6	8	0.5	16	0.55

**Table 3:** Travel mode by staff/student status.

	Motor car (%)	Public transport ^1^ (%)	Active Travel ^2^ (%)
**Total (n = 2,744)**	**26.6**	**49.8**	**23.6**
staff (n = 1,221)	37.7	36.3	26.0
students (n = 1,561)	18.0	60.3	21.7
**Age**			
<25 years (n = 1,107)	15.7	67.1	17.2
25–44 years (n = 1,054)	28.3	40.5	31.2
45 + years (n = 2,744)	44.1	33.8	22.1
**Sex**			
Male (n = 912)	23.4	46.9	29.7
Female (n = 1,833)	28.2	51.2	20.6
**Education**			
No degree (n = 1,088)	18.8	65.5	15.6
Degree (n = 1,242)	29.6	43.3	27.1
PhD (n = 437)	37.1	29.3	33.6
**Self-reported physical activity**			
Insufficiently active (<150 min/week) (n = 1,541)	28.8	47.3	23.9
Sufficiently active (150 + min/week) (n = 948)	24.3	54.0	21.7
**Total minutes of self-reported physical activity**			
Mean in previous week	141.9	158.6	143.6

**^1^** = train and bus; **^2^** = walking, cycling, skateboard or scooter.

### 3.4. Walking

490 people walked to a University campus, representing 16.9% of travel mode. Walkers were more like to be female (64.9%), be a student (54.3%), be aged 25–44 years (46.4%), and have a degree (51.8%).

### 3.5. Public Transport Use

443 staff and 941 students used public transport as their main mode of travelling to the University on the day of the survey representing a 36.3% and 60.3% share to public transport. Students, as a result of their income and because of their transient lifestyle at University, are typically high public transport users [33]. Overall, public transport users were more likely to be under 25 years old (67.1%), female (51.2%) and not have a degree (65.5%).

### 3.6. Factors Associated with Mode Use

Logistic regression was used to identify the associations between users of different modes of travel and a range of independent variables. Table 4 highlights that active commuters (walking and cycling only) were 71% more likely to be students, 33% less likely to be female, less likely to be older than 25, but education level was not significant. When the definition of active travel excludes public transport, active travellers were 44% more likely to be students, 33% less likely to be female, 61% more likely to be aged 25–45 years and more likely to have a degree or higher level of education.

### 3.7. Physical Activity

Overall, 41% of respondents were sufficiently active (defined by meeting physical activity recommendations of 150 min per week) [34]. Participants were more likely to meet physical activity recommendations if they travelled actively. Specifically, 46% of active travellers (not including public transport users) were sufficiently active compared to 39% of motor vehicle and public transit users. When public transport use was included in the definition of active travel 44% of active travellers and public transit users were sufficiently active compared to 31% of motor vehicle users.

The average total number of minutes spent in self-reported physical activity was 154 min (SD 212.5) per week. Students (164 min) reported they were more active than staff (139 min) which was consistent with younger respondents (172 min) being more active than those older than 25 years (142 min). In this sample women (159 min) were more active than men (142 min). Those people who walked or cycled to University achieved a mean of 189 min of self-reported physical activity per week, compared to 149 min for those non-active travellers. If public transport is included in the definition of active travel, the average total minutes of self-reported physical activity is 174 min per week, compared to 115 min for the least active travellers.

In logistic regression models (see Table 5) looking at factors associated with achieving adequate physical activity, active travel (both with (AOR = 1.71, 95% CI 1.41–2.07) or without (AOR = 1.34, 95% CI 1.10–1.62) public transport) was significantly associated with achieving recommended levels of self-reported physical activity after adjustment for staff-student status, age, sex and education.

**Table 4:** Multivariate model * of odds of being an active traveller (walking and cycling only) compared with motor vehicle travel and public transport combined, plus active travel including public transport compared with motor vehicle travel, for staff and students **.

		Active travel (walking and cycling only)					Active travel including public transport				
	N	% active travel	Odds ratio	95% CI	Adjusted odds ratio	95% CI	% active travel	Odds ratio	95% CI	Adjusted odds ratio	95% CI
Staff	1,221	26.0	1.0		1.0		62.3	1.0		1.0	
Students	1,561	21.7	0.79	0.66–0.94	1.43	1.12–1.85	82.0	2.75	2.32–3.27	1.71	1.33–2.20
Males	912	29.7	1.0		1.0		76.6	1.0		1.0	
Females	1,833	20.6	0.61	0.51–0.74	0.67	0.55–0.81	71.9	0.78	0.65–0.93	0.67	0.55–0.81
Under 25 years	1,426	17.2	1.0		1.0		84.3	1.0		1.0	
25–44 years	1,405	31.2	2.19	1.79–2.68	1.61	1.21–2.14	71.7	0.47	0.38–0.58	0.65	0.48–0.87
45+ years	752	22.1	1.37	1.07–1.76	1.05	0.74–1.49	55.9	0.24	0.19–0.30	0.37	0.26–0.51
No degree	1,088	15.6	1.0		1.0		81.2	1.0		1.0	
Degree	1,242	27.1	2.00	1.63–2.46	1.82	1.41–2.35	70.4	0.55	0.45–0.67	0.94	0.73–1.20
PhD	437	33.6	2.73	2.12–3.54	2.74	1.94–3.86	62.9	0.39	0.31–0.50	0.95	0.69–1.30

* Shading indicates statistically significant association; ** The Hosmer–Lemeshow test of goodness of fit excluding public transport (*p* < 0.001); The Hosmer–Lemeshow test of goodness of fit including public transport (*p* = 0.070).

**Table 5:** Adjusted model * of odds of being adequately physically active and travel mode for walking and cycling only compared with motor vehicle travel, and public transport combined with walking and cycling **.

		Adequate physical activity				
	N	% adequatelyactive ***	Odds ratio	95% CI	Adjusted odds ratio	95% CI
Motor vehicle travel	1,909	39.2	1.0		1.0	
Active travel excluding public transport	574	45.6	1.30	1.08–1.57	1.33	1.10–1.62
Motor vehicle travel	657	31.4	1.0		1.0	
Active travel including public transport	1,826	44.1	1.73	1.43–2.09	1.71	1.41–2.07

* Adjusting for staff/student status, age, sex and education; ** The Hosmer–Lemeshow test of goodness of fit excluding public transport (*p* = 0.920); The Hosmer-Lemeshow test of goodness of fit including public transport (*p* = 0.293); *** Adequate physical activity refers to more than 150 min of moderate-to-vigorous physical activity in the past 7 days.

## 4. Discussion

These results indicate that including public transport users in the definition of active transport both substantially increases the proportion of respondents considered to be actively travelling but also the likelihood of achieving recommended levels of physical activity. While walkers and cyclists achieved a higher total mean minutes of weekly self-reported physical activity, when public transport users were added to this group, there was only a small reduction in the mean total weekly minutes of self-reported physical activity. From a public health perspective, because public transport users include a large proportion of respondents, the overall public health benefit of active travel is greater if public transport is included as part of the definition of active travel.

Parking strategies form the mainstay of many travel plans since inexpensive and convenient parking is known to be associated with increased likelihood of using private motor vehicles for commuting [35,36]. In this survey, approximately 10% of car user respondents identified using free car parking on the campus. Increases in parking costs for the majority using paid parking would directly impact upon decisions about travel mode choice [35]. As with other travel plans, if increasing the cost and reducing the convenience of parking should be a feature of the University travel plan, the survey response suggest that this would affect the cost for a small but not insignificant proportion of people which in turn is likely to influence their travel mode choice.

The student travel mode behaviour is broadly consistent with an (unpublished) convenience sample intercept survey conducted at Sydney University during Orientation Week in 2004. In this survey, of 587 students who completed a brief questionnaire, 35% walked or cycled, and a further 53% used public transport to get to the University. The 2006 Census journey to work data were examined for employed persons travelling to the Camperdown campus. These data, also unpublished, indicate that 42% of staff used a motor vehicle to travel to work—a figure consistent with data reported here.

University staff members were twice as likely as students to drive to a campus, suggesting that efforts to change travel behaviour away from motor vehicles would be best directed towards staff. The higher prevalence of driving to work among staff may be a function of age and life situations associated with age (e.g., having children, not living close to campus).

For students, increasing active travel may need to be focused on short trips where walking or cycling is feasible. This may mean a reduction in public transport use which in turn would release much needed capacity in Sydney. However, rates of walking, cycling and public transport use combined were higher compared with students elsewhere in Australia: at the University of Western Australia, for example, 46.8% of students regularly used these active modes [20]. This may be a function of different contexts and available public transport and walking and cycling infrastructure, and the variation suggests that with some targeted programs, rates of student walking and cycling can be increased.

The results of this survey have indicated that higher levels of education were associated with walking and cycling to University. This is consistent with previous research using NSW Health Survey data that showed, after adjusting for residence and socio-economic status, having a University degree or higher was associated with more frequent walking and cycling to work across New South Wales, Australia [23]. However, in the survey here, education level becomes non-significant when active travellers include users of public transport, probably of the wide diversity of people that use public transport to access the University, including support staff with typically lower education levels. More information about the benefits of walking and cycling plus information on how it could feasibly become part of the journey to the University could increase rates of active travel.

Levels of self-reported physical activity in the surveyed sample were somewhat lower than population levels, with only 41% achieving recommended levels. NSW Population Health Survey data indicate that in 2011, 59.6% of men and 48.5% of women in NSW aged 16 years and over were adequately physically active [37]. While this is an important finding, and supports the need for a healthy University initiative which aims to increase physical activity, further assessment of the prevalence of adequate physical activity is needed because the sample may not have been representative of the University population.

Most respondents (80%) travelled to the Camperdown Campus, with Cumberland Campus the next most frequent destination (8%) and hence the results of this report are most applicable to the Camperdown campus. More women responded to the survey, as compared to the University population profile. With the results showing women are less likely than men to be active travellers suggesting the sample results for active travel modes may be conservative estimates for the population.

This survey represents important planning and evaluation information for the “Move more—sit less” Healthy University working group. For example, the finding that 40% of respondents lived within 5 km of the campus, and 39% lived between 5 and 15 km from the University, helps shape realistic targets in terms of what might be achievable in terms of shifts to active travel. The higher level of driving by staff suggests that carrot and stick strategies such as discounted annual travel passes coupled with restrictions on parking or increased parking costs might be effective. High levels of public transport use by students, particularly trains which involve a relatively long walk to lecture rooms, may mean cycling skills and promotion programs may be effective [38].

Despite technical difficulties, there was a good response to the survey resulting in 3,737 usable responses. However this represents a relatively low overall response rate from over 45,000 students, not unusual for on-line surveys [39,40]. The exact response rate is impossible to calculate as it is not known how many of the staff or students were part time or may not have received the e-mail and survey link with sufficient time to respond. The limitations of the study also include a greater than expected degree of missing data, a disproportionately high female response, and some lack of representation from all Faculties. Nevertheless, the survey provides baseline information more focused on the University for the development of the Green Travel Plan and strategies to increase active travel and physical activity for members of the University. It is intended that the survey will be repeated in 3–5 years, to monitor the progress following implementation of strategies to increase active travel and physical activity. Findings from the current baseline survey and the evaluation of the improvement in travel plans at the University of Sydney will inform other similar programs in universities and other employment settings.
